# Bringing question notes to physicians: A nationwide cross-sectional study in Japan (INFORM study 2020)

**DOI:** 10.1016/j.pecinn.2025.100398

**Published:** 2025-04-30

**Authors:** Mariko Asai, Masako Okamura, Maiko Fujimori, Aki Otsuki, Junko Saito, Akiko Yaguchi-Saito, Aya Kuchiba, Yosuke Uchitomi, Taichi Shimazu, for the INFORM Study Group

**Affiliations:** a Division of Survivorship, National Cancer Center Institute for Cancer Control, National Cancer Center, Tokyo, Japan; b Department of Medical Psychology, Nippon Medical School, Tokyo, Japan; c Faculty of Pharma-Sciences, Teikyo University, Tokyo, Japan; d Division of Behavioral Sciences, National Cancer Center Institute for Cancer Control, National Cancer Center, Tokyo, Japan; e Division of Prevention, National Cancer Center Institute for Cancer Control, National Cancer Center, Tokyo, Japan; f Faculty of Human Sciences, Tokiwa University, Mito, Japan; g Division of Biostatistical Research, Institution for Cancer Control/ Biostatistics Division, Center for Administration and Support, National Cancer Center, Tokyo, Japan; h Graduate School of Public Health, Teikyo University, Tokyo, Japan; i Department of Cancer Survivorship and Digital Medicine, The Jikei University School of Medicine, Japan

**Keywords:** Bringing question notes, Nationwide cross-sectional study, INFORM study 2020, Japan

## Abstract

**Objective:**

To describe the behavior of bringing question notes to physicians in Japan and explore the factors associated with this behavior.

**Methods:**

We used data from a nationwide cross-sectional study conducted in Japan on access to health information (INFORM Study 2020). Data from 3605 respondents, 3572 of whom did not miss the primary endpoint of bringing question notes to physicians, were analyzed. The prevalence of weighted ratios of four-item responses (always, usually, sometimes, never) was described, and multivariable logistic regression was used to explore associated variables with “never bringing question notes.” We also explored the differences in these factors based on the cancer diagnosis experience.

**Results:**

More than 60 % of all respondents and even among those who had a history of cancer diagnosis themselves, more than 50 % had never brought question notes to physicians. Being under 40 years of age, male, employed, and in good health were significantly associated with never bringing question notes.

**Conclusion:**

Those who had never brought question notes to physicians were high both overall and among those who had a cancer diagnosis.

**Innovation:**

Nationwide survey data revealed that Japanese people are less proactive in bringing the notes prepared beforehand to the consultation.

## Highlights

• A nationwide cross-sectional survey on health information in Japan was conducted.
• This study describe the behavior of bringing question notes to physicians.
• More than 60 % of all respondents had never brought question notes to physicians.
• Among those with a history of cancer diagnosis, more than half had never brought.

## 1 Introduction

Good communication between physicians and patients increases patient adherence [1] and positively affects patients' health status [2]. Asking questions increases patient self-efficacy, leading to a positive turnaround in health status [3]. Physicians who provide more information are considered more cooperative and a partner to patients, who are encouraged to engage in proactive communication behaviors [4]. Asking questions is a health behavior that should be recommended to patients when consulting a physician [3].

Cancer care involves many decision-making situations and requires good communication between physicians and patients [5]. The effectiveness of using a question prompt list (QPL) to facilitate questions for physicians has been reported in a systematic review [6], and its usefulness has also been reported in Japan [7]. In the communication guidelines for cancer treatment published in recent years in Japan, QPL is recommended for use owing to its high level of supportive evidence [8]. The behavior of bringing question notes to physicians is considered a preparatory step for patients to proactively ask their physicians questions, however, no study has elucidated this behavior.

For the first time, we conducted a nationally representative cross-sectional survey on health information access among consumers in Japan in 2020 (INFORM Study 2020) [9]. This study was designed to strengthen health behaviors for cancer prevention in Japan, drawing on the Health Information National Trends Survey (HINTS) conducted in the United States [10,11] that began in 2003. In this study, we report the behavior of bringing question notes to physicians, which was adopted as a question in a previous HINTS study. The reason we are focusing on this item this time is that in a survey of the intentions of Japanese cancer patients that we conducted previously, we found that many patients wanted their doctors to prompt them to ask questions [12], and in fact, when QPL was actually used, the number of questions asked by Japanese patients was low [7]. Furthermore, it was not clear whether the questions were clear and prepared but difficult to ask the doctor, or whether the questions were not yet clear, so it was thought that it would be desirable to educate patients about the use of QPL after clarifying what stage of preparation they were at.

Therefore, the aim of this study was to clarify the actual situation of patients bringing their question notes to consultations as an indicator of the state in which patients have clarified what they want to ask and are preparing for the consultation, as a preliminary step to asking questions of the doctor. Furthermore, as mentioned above, there are many decision-making situations in cancer treatment, and patients and their families are often faced with difficult treatment choices. Therefore, the aim of this study was to analyze whether the frequency of the action of “bringing” differs depending on whether the person or their family has experienced cancer treatment. [9]

## 2 Methods

### 2.1 Study design and respondents

Data were drawn from the INFORM Study 2020, a nationally representative cross-sectional mail survey conducted using a self-administered questionnaire in 2020. INFORM Study 2020 sampled 10,000 Japanese aged over 20 years using two-stage stratified random sampling, with the census area as the primary sampling unit and individuals aged 20 years or older as the secondary sampling unit. Details of the sampling strategy used for this national survey are described in a protocol paper [9]. From the 35 strata, we crossed nine regions and four municipal groups by population size to randomly select 500 census areas with a probability proportional to the stratum size. Ethical approval for this study was granted by the Research Ethics Committee of the National Cancer Center (research project number: 2019–290 for the national survey).

### 2.2 Measures

#### 2.2.1 “Bringing question notes to physicians”

The data were evaluated on one item: “Generally, how often do you take a list of questions to your physician visits?” Respondents were asked to select from the following four options: *always*, *usually*, *sometimes*, and *never*. This item has only been used in Health Information National Trends Survey in the United States, called HINTS4Cycle3 (2013) [10].

#### 2.2.2 Sociodemographic and health-related variables

Sociodemographic variables included in this study were age, sex, education, marital status, employment status, and annual income. The analysts involved in this study performed the categorization of variables. Further, health-related variables included health-related perceptions, which were assessed using the following two items: 1) general health status rated on a five-point Likert scale (excellent, very good, good, fair, and poor) and 2) confidence in health management rated on a five-point Likert scale (completely, very, somewhat, a little, and not at all). History of cancer diagnosis was classified into three categories: 1) personal history that includes those who have both their own diagnosis and that of a family member, 2) family history only, and 3) no history that includes those who have never had a personal or family history or whose family history is unknown.

### 2.3 Statistical analyses

To estimate accurate parameter estimates for the Japanese general population, we conducted a weighted analysis to account for the complex sampling design and missing responses [9]. We calculated confidence intervals (CI) using the Taylor series linearization method [13]. All analyses were conducted using IBM SPSS Statistics for Windows, version 29.0, with a complex sampling model (IBM Corp, Armonk, NY, USA). In the HINTS reference data, missing-value exclusion and cancer diagnosis classification were performed using the same procedure as in the present study.

Descriptive statistics were calculated using weighted analyses of all the sociodemographic and health-related variables. We further conducted a subgroup analysis based on the history of cancer diagnosis: 1) personal history, including those with their own diagnosis and that of a family member; 2) family history only; and 3) no history, including those who had never had a personal or family history or whose family history is unknown. For the association of the variables with never bringing question notes, we used a multivariable logistic regression model adjusted for all sociodemographic and health-related variables (age, sex, education, marital status, employment status, annual income, general health status, confidence in health management). Statistical significance was set as a *p*-value of <0.05.

## 3 Results

### 3.1 Respondents characteristics

**Fig. 1:**
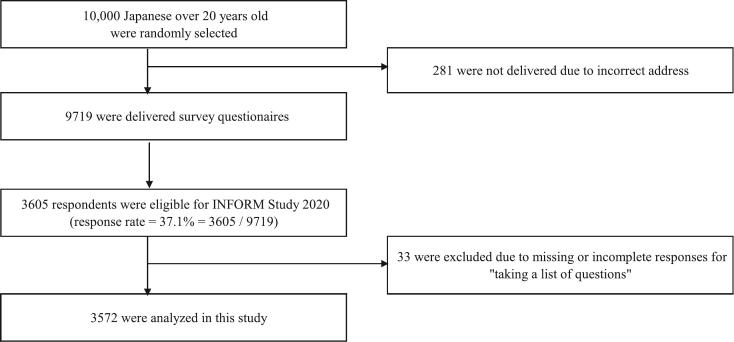
Study population.

Using the INFORM Study 2020 data, we reached out to 9719 of 10,000 individuals identified in the sampling. Totally, 3605 responded (response rate = 3605/9719 = 37.1 %). After excluding 33 participants with missing data for the dependent variable of “bringing question notes to physicians,” we analyzed the data of 3572 respondents (Fig. 1).

Table 1 shows the sociodemographic and health-related characteristics of the respondents. The mean age at the time of the survey was 56 years (SD: 17 years; range: 20–97 years). [11]

**Table 1:** Respondent Characteristecs. (*n* = 3572).

Characteristics	n	Weighted %	95 %CI
Age (years)			
20–39	696	23.6	22.0–25.4
40–64	1629	42.1	40.3–44.0
65 +	1247	34.2	32.4–36.1
Gender			
Male	1628	49.1	47.5–50.8
Female	1944	50.9	49.2–52.5
Education			
High school graduate or lower	1694	48.1	46.1–50.0
Some college	826	21.7	20.5–23.0
College graduate or above	1040	30.2	28.4–32.0
Marital status			
Married	2503	67.2	65.4–69.0
Not married	1056	32.8	31.0–34.6
Employment status			
Employed	2243	62.8	60.9–64.6
Unemployed	472	14.4	13.1–15.8
Retired / Homemaker / Student	843	22.8	21.3–24.3
Annual income(Japanese yen)			
< 4 million	1310	37.8	35.9–39.8
< 8 million	1364	39.0	37.2–40.8
8 million +	794	23.2	21.6–24.9
General health status			
Excellent / Very good / Good	2705	76.0	74.3–77.5
Fair / Poor	846	24.0	22.5–25.7
Confidence in health management			
Completely / Very / Somewhat	1904	53.6	51.8–55.3
A little / Not at all	1647	46.4	44.7–48.2
History of cancer diagnosis			
No history⁎	1525	43.9	42.2–45.7
Family history only	1660	45.7	44.0–47.5
Personal history	387	10.4	9.30–11.5

⁎ Include family history unknown.

### 3.2 Prevalence of “bringing question notes to physicians”

As shown in Table 2, the highest percentage of respondents (64.1 %) answered that they had never brought a question memo to their physician. Among those with a history of cancer diagnosis, more than half had never brought.

**Table 2:** Prevalence of “bringing question notes to physicians”.

Response rate = 37.1 %	Overall		Personal history		Family history only		No history⁎	
	(n = 3572,100 %)		(*n* = 387, 10.4 %)		(*n* = 1660, 45.7 %)		(*n* = 1525, 43.9 %)	
	weighted %	95 % CI	weighted %	95 % CI	weighted %	95 % CI	weighted %	95 % CI
Always	2.80	2.30–3.40	4.80	3.00–7.50	2.60	1.80–3.50	2.50	1.80–3.50
Usually	6.90	6.10–7.80	10.1	7.30–13.7	7.20	6.00–8.60	5.80	4.70–7.20
Sometimes	26.2	24.8–27.7	34.4	29.3–39.1	25.7	23.7–27.9	24.9	22.7–27.2
Never	64.1	62.3–65.8	51.1	45.8–56.5	64.4	62.0–66.8	66.8	64.3–69.2

⁎ Includes family history unknown.

### 3.3 Associated variables with “*never* bringing question notes to physicians”

As shown in Table 3, the adjusted odds ratios for significantly associated items (*p* < .01) among the respondents were as follows. Age (reference: 20–39 years) was significant at 0.67 (95 % CI: 0.53–0.84) for those aged 40–64 years and 0.45 (95 % CI: 0.35–0.59) for those aged 65 years and older. Sex (reference: male) was significant at 0.60 (95 %CI: 0.51–0.70) for female respondents. Employment (reference: employed) was significant at 0.70 (95 %CI: 0.53–0.91) for unemployed respondents and 0.73 (95 %CI: 0.60–0.88) for retired or homemaker or student respondents. Health (reference: person in good health) was also significant at 0.58 (95 %CI: 0.48–0.69) for those in poor health.

**Table 3:** Association of respondent characteristics with “ Never bringing question notes to physicians.”

		Overall		Cancer status group					
				Personal history		Family history only		No history⁎	
Characteristics		adujusted OR	95 %CI	adujusted OR	95 %CI	adujusted OR	95 %CI	adujusted OR	95 %CI
Age (years)		P < .01		*P* = .42		P < .01		P < .01	
	20–39	Reference		Reference		Reference		Reference	
	40–64	**0.67**	**0.53–0.84**	0.46	0.10–2.09	**0.65**	**0.47–0.88**	**0.66**	**0.47–0.93**
	65 +	**0.45**	**0.35–0.59**	0.36	0.07–1.80	**0.43**	**0.30–0.61**	**0.45**	**0.30–0.68**
Gender		P < .01		*P* = .03		P < .01		P < .01	
	Male	Reference		Reference		Reference		Reference	
	Female	**0.60**	**0.51–0.70**	0.58	0.35–0.94	**0.52**	**0.41–0.66**	**0.65**	**0.51–0.83**
Education		*P* = .01		P = .10		P = .03		*P* = .37	
	High school graduate or lower	Reference		Reference		Reference		Reference	
	Some college	0.85	0.70–1.03	0.48	0.25–0.94	0.91	0.68–1.20	0.87	0.64–1.17
	College graduate or above	0.76	0.63–0.91	0.85	0.48–1.50	0.68	0.52–0.91	0.82	0.62–1.09
Marital status		*P* = .51		*P* = .87		*P* = .16		*P* = .68	
	Married	Reference		Reference		Reference		Reference	
	Not married	1.06	0.89–1.26	1.05	0.60–1.82	1.19	0.93–1.52	0.94	0.70–1.26
Employment status		P < .01		*P* = .75		*P* = .14		*P* = .02	
	Employed	Reference		Reference		Reference		Reference	
	Unemployed	**0.70**	**0.53–0.91**	0.81	0.41–1.61	0.73	0.50–1.07	0.66	0.43–1.02
	Retired / Homemaker / Student	**0.73**	**0.60–0.88**	1.01	0.58–1.76	0.79	0.60–1.05	0.64	0.46–0.89
Annual income (Japanese yen)		*P* = .73		*P* = .24		*P* = .50		P = .02	
	< 4 million	Reference		Reference		Reference		Reference	
	4 million <8 million	0.94	0.78–1.14	0.89	0.54–1.45	1.17	0.89–1.54	0.73	0.54–0.99
	8 million +	0.92	0.74–1.14	1.74	0.79–3.84	1.17	0.84–1.62	0.62	0.44–0.88
General health status		P < .01		*P* = .20		P < .01		P = .01	
	Excellent / Very good / Good	Reference		Reference		Reference		Reference	
	Fair / Poor	**0.58**	**0.48–0.69**	0.72	0.43–1.20	**0.46**	**0.35–0.59**	0.69	0.52–0.93
Confidence in health management		*P* = .22		*P* = .54		*P* = .09		*P* = .88	
	Completely / Very / Somewhat	Reference		Reference		Reference		Reference	
	A little / Not at all	1.11	0.94–1.30	1.16	0.71–1.90	1.24	0.97–1.59	0.98	0.76–1.26
History of cancer diagnosis		P = .06							
	No history⁎	Reference							
	Family history only	0.96	0.82–1.12						
	Personal history	0.73	0.57–0.95						
Time since diagnosis (years)				P = .10					
	1 or less			Reference					
	2–5			1.22	0.60–2.46				
	6+			1.77	0.98–3.20				

Multivariable logistic regression model adjusted for all variables (age, gender, education, marital status, employment status, annual income, general health status, confidence in health management).

Statstic significance indicated by bold hilights (p < .01).

⁎ Include family history unknown.

Moreover, the variable of history of cancer diagnosis was significant at 0.73 (95 % CI: 0.57–0.95) for own diagnosis and 0.96 (95 %CI 0.82–1.12) for family members when “none” was used as a reference. We found no significant difference (*p* = .06). Furthermore, if the respondent had a personal history of cancer diagnosis, the longer the time since diagnosis, the less likely they were to bring a memo, but the association was not significant (*p* = .10).

## 4 Discussion and conclusion

### 4.1 Discussion

This study revealed, for the first time, the status of Japanese adults regarding the behavior of bringing question notes to physicians, based on analysis of data from a nationwide survey.

Respondents who had never brought question notes to physicians accounted for the most significant proportion, at more than 60 %, and even among those who had a history of cancer diagnosis themselves, more than 50 % had never brought question notes to physicians. Conversely, according to the results of the HINTS 2013, which we analyzed as a reference (see supplementary material), 30 % of all respondents in the US never brought notes. Furthermore, less than 20 % of those who had a history of cancer diagnosis and 30 % of those who always brought notes were the most common. However, a simple comparison between Japan and the US is not possible based on our study and the HINTS data. Nonetheless, the Japanese samples tended not to bring notes with them during medical consultations, even those with first-hand experience of a cancer diagnosis—that is, after seeing a physician as a patient with cancer. This lack of proactive behavior in taking notes may be related to patients' passive attitude toward wanting physicians to prompt them to ask questions, as revealed in a previous study [12].

Personal attributes, such as being under 40 years old, male, employed, and in good health, were significantly associated with those who had never engaged in the behavior of bringing question notes. As this study did not investigate the reasons for not bringing notes, we cannot comment on the background of these related factors. Nevertheless, we must educate and inform the general public that bringing notes is an easy way to communicate needs to doctors, and that notes encourage doctors to provide more information and be more cooperative (4). In fact, education is necessary to encourage patient-centered behavior.

Our findings showed that Japanese people are unlikely to bring a list of questions to prepare for asking the physician questions, and this was also the case even if they themselves had been diagnosed with cancer. These results are also related to the results of our previous survey [12], which showed that Japanese patients with cancer have a need for their physicians to prompt them with questions, which is a characteristic of patients with cancer in Japan compared to those in the US. QPLs, as tools that doctors can use to encourage patients to ask questions, may be obtained freely, such as the one currently available for download from the website of the Cancer Information Service run by the National Cancer Centre of Japan [14]. This QPL needs to be recommended to more Japanese people.

Finally, although it is important to evaluate patient indicators as an effect of the behavior of asking questions to doctors by bringing question note, this study did not use such indicators, so this remains as future tasks. In previous Japanese research [7], although usefulness increased significantly, there was no significant intervention effect on satisfaction with the consultation, and furthermore, a systematic review also showed that there was no significant intervention effect on patient satisfaction with the consultation [6]. In these studies, usefulness is evaluated in terms of the QPL material, and satisfaction is evaluated in terms of the doctor's consultation. As for the clinical significance of preparing questions before the consultation, it may be worth considering using the patient's own cognitive indicators, such as motivation for treatment and self-esteem.

The following limitations were present in this study. First, the response rate for our survey was 37 %. This response rate is almost the same as that for the HINTS 2013 survey in the US, and although we adjusted for the non-response bias using weighting, we could not rule out the possibility of a limited generalizability of our results. Second, the survey items were limited; because it only referred to the HINTS 2013 data in the US, further research is needed to compare the patient interview behavior between Japan and the US. Third, our study assumed the context of the general consultation and may not apply to major medical examinations, such as decision-making in cancer treatment. Lastly, in this cross-sectional study, we could not identify causal relations.

### 4.2 Innovation

Nationwide survey data revealed that Japanese people are less proactive in bringing their questioning notes. The need to actively educate patients on questioning physicians was indicated, as bringing questioning notes to consultations and showing them to physicians could catalyze QPL use and patient-centered communication.

### 4.3 Conclusion

This study showed that those who had never brought question notes to physicians were high in both the overall sample and among those who had a cancer diagnosis. Along with educating cancer patients to take the initiative in asking questions, health authorities should recommend the use of QPLs as a tool to assist patients with cancer patients in asking questions to their physicians.

## Authors' contribution

TS, AO, JS, AY, AK, MF, and YU conceptualized and developed the study design and assessments. MA and MF analyzed and interpreted the data under the supervision of AK. MA wrote the manuscript outline, which other authors carefully revised, edited, and discussed. All the authors have read and approved the final version of the manuscript.

## CRediT authorship contribution statement

**Mariko Asai:** Writing – original draft, Methodology, Formal analysis, Data curation. **Masako Okamura:** Writing – review & editing, Supervision, Formal analysis, Data curation. **Maiko Fujimori:** Writing – review & editing, Writing – original draft, Visualization, Validation, Supervision, Software, Resources, Project administration, Methodology, Investigation, Formal analysis, Data curation, Conceptualization. **Aki Otsuki:** Writing – review & editing, Supervision, Project administration, Methodology, Investigation, Formal analysis, Data curation, Conceptualization. **Junko Saito:** Writing – review & editing, Supervision, Methodology, Investigation, Data curation, Conceptualization. **Akiko Yaguchi-Saito:** Writing – review & editing, Methodology, Conceptualization. **Aya Kuchiba:** Writing – review & editing, Methodology, Data curation. **Yosuke Uchitomi:** Writing – review & editing, Supervision, Project administration, Methodology, Investigation, Conceptualization. **Taichi Shimazu:** Writing – review & editing, Writing – original draft, Visualization, Validation, Supervision, Software, Resources, Project administration, Methodology, Investigation, Funding acquisition, Formal analysis, Data curation, Conceptualization.

## Ethics approval and consent to participate

Ethical approval for this study was granted by the Research Ethics Committee of the National Cancer Center (research project numbers: 2019–290 for the national survey).

## Declaration of competing interest

The authors declare that they have no known competing financial interests or personal relationships that could have appeared to influence the work reported in this paper.

## Data Availability

The data sets used in this study are not publicly available. However, anonymized datasets may be made available after approval by the INFORM Study group and the institutional review board. Proposals for the use of data (research question, aim, background, design, and analytical plan) should be submitted to the corresponding author, M. F.

## Appendix A Supplementary data

Supplementary material Image 1
